# Supplementary material for: Novel Driver Strength Index highlights important cancer genes in TCGA PanCanAtlas patients
Source: PeerJ. 2022 Aug 11;10:e13860. doi: 10.7717/peerj.13860 (PMC9375969; doi:10.7717/peerj.13860)
Supplement: Supplemental Information 2 [file peerj-10-13860-s002.zip › COHORTS/genes plots/detailed/2021_8_16_14_9_distribution_events_detailed_males_22.pdf]

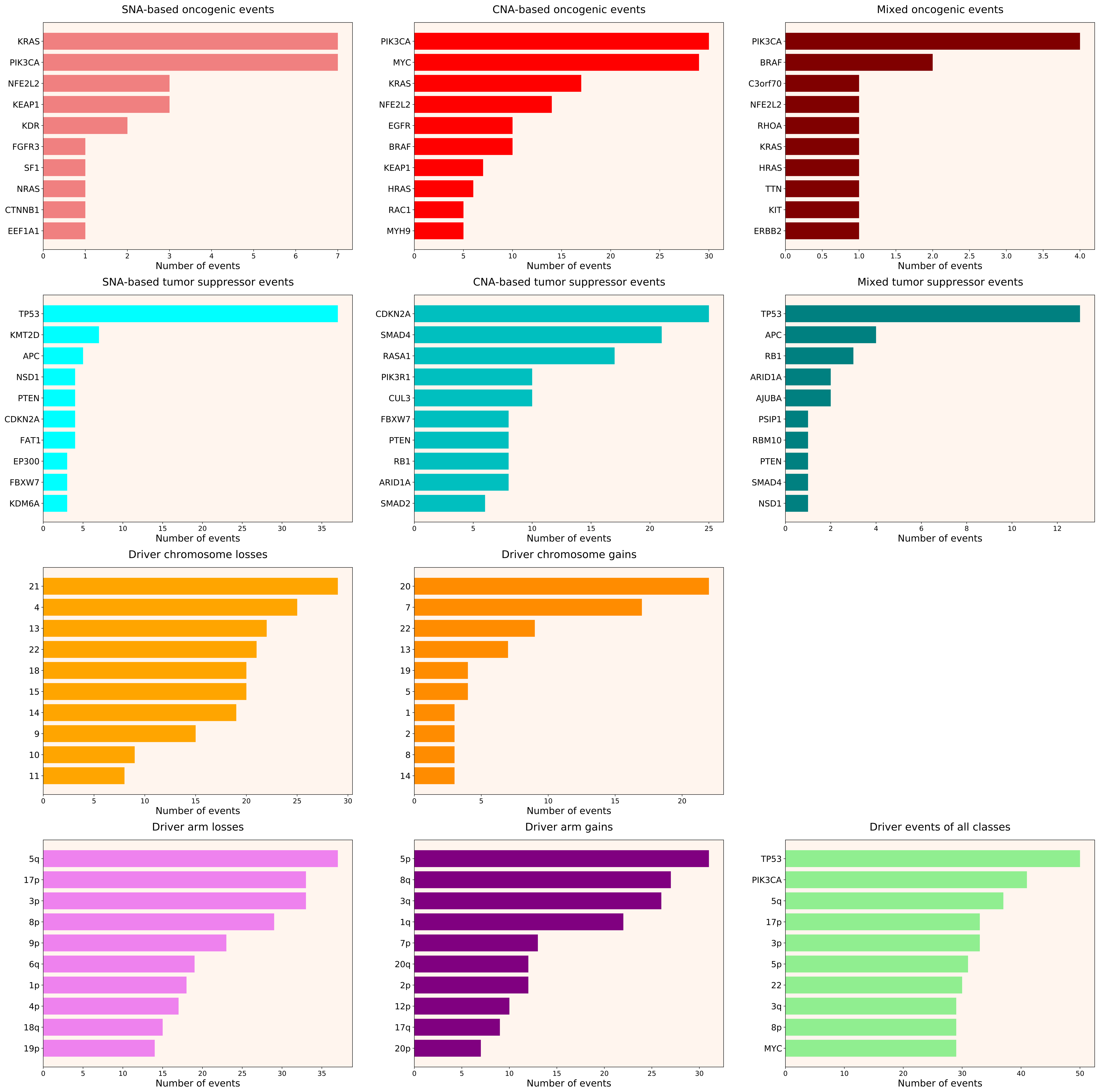

SNA-based oncogenic events
KRAS
PIK3CA
NFE2L2
KEAP1
KDR
FGFR3
SF1
NRAS
CTNNB1
EEF1A1
Number of events
CNA-based oncogenic events
PIK3CA
MYC
KRAS
NFE2L2
EGFR
BRAF
KEAP1
HRAS
RAC1
MYH9
Number of events
Mixed oncogenic events
PIK3CA
BRAF
C3orf70
NFE2L2
RHOA
KRAS
HRAS
TTN
KIT
ERBB2
Number of events
SNA-based tumor suppressor events
TP53
KMT2D
APC
NSD1
PTEN
CDKN2A
FAT1
EP300
FBXW7
KDM6A
Number of events
CNA-based tumor suppressor events
CDKN2A
SMAD4
RASA1
PIK3R1
CUL3
FBXW7
PTEN
RB1
ARID1A
SMAD2
Number of events
Mixed tumor suppressor events
TP53
APC
RB1
ARID1A
AJUBA
PSIP1
RBM10
PTEN
SMAD4
NSD1
Number of events
Driver chromosome losses
21
4
13
22
18
15
14
9
10
11
Number of events
Driver chromosome gains
20
7
22
13
19
5
1
2
8
14
Number of events
Driver arm losses
5q
17p
3p
8p
9p
6q
1p
4p
18q
19p
Number of events
Driver arm gains
5p
8q
3q
1q
7p
20q
2p
12p
17q
20p
Number of events
Driver events of all classes
TP53
PIK3CA
5q
17p
3p
5p
22
3q
8p
MYC
Number of events
